# A Systems Thinking Approach to Inform Coherent Policy Action for NCD Prevention: Comment on "How Neoliberalism Is Shaping the Supply of Unhealthy Commodities and What This Means for NCD Prevention"

**DOI:** 10.15171/ijhpm.2019.113

**Published:** 2019-11-16

**Authors:** Penelope Milsom, Richard Smith, Helen Walls

**Affiliations:** ^1^Department of Global Health and Development, Faculty of Public Health and Policy, London School of Hygiene and Tropical Medicine, London, UK.; ^2^College of Medicine and Health, University of Exeter, Exeter, UK.

**Keywords:** Neoliberalism, Policy Coherence, Non-communicable Disease Prevention, Complex Systems

## Abstract

Lencucha and Thow tackle the enormous public health challenge of developing non-communicable disease (NCD) policy coherence within a world structured and ruled by neoliberalism. Their work compliments scholarship on other causal mechanisms, including the commercial determinants of health, that have contributed to creating the risk commodity environment and barriers to NCD prevention policy coherence. However, there remain significant gaps in the understanding of how these causal mechanisms interact within a whole system. As such, public health researchers’ suggestions for how to effectively prevent NCDs through addressing the risk commodity environment tend to remain fragmented, incomplete and piecemeal. We suggest this is, in part, because conventional policy analysis methods tend to be reductionist, considering causal mechanisms in relative isolation and conceptualizing them as linear chains of cause and effect. This commentary discusses how a systems thinking approach offers methods that could help with better understanding the risk commodity environment problem, identifying a more comprehensive set of effective solutions across sectors and its utility more broadly for gaining insight into how to ensure recommended solutions are translated into policy, including though transformation at the paradigmatic level.

Lencucha and Thow’s^1^ analysis begins to reveal the risk commodity environment as a complex systems problem, in which interactions between multiple elements and agents across political, economic and social domains mean that decisions taken at one level can have multiple, often delayed and distant, effects elsewhere.^2^ However, they do not progress this formulation, critically the role of dynamic complexity – the often unintuitive behaviour of a complex system that arises from interaction of elements and agents over time – that we suggest underlies the political challenge of achieving greater policy coherence for non-communicable disease (NCD) prevention.^2^

While there is no single accepted definition of a systems thinking approach, broadly speaking system methods ‘enable researchers and decision-makers to examine system components, and the dynamic relationships between them, at multiple levels, from cell to society.’^3^ The utility of this approach for understanding the etiology of NCDs is increasingly recognized.^4^ In considering the dynamic complexity of complex systems, Sterman usefully describes a set of characteristics that would apply well to various NCD policy challenges that have emerged out of a dominant neoliberal paradigm.^2^ For Sterman, complex systems display:

- Constant change at different time scales. For example, political support for alcohol harm reduction policies may have been building over time, then the election of a more commercially-orientated president may abruptly reverse this progress.
- Tight coupling of actors and elements within the system such that any action generates multiple reactions with both predictable and unintended impacts. Reduced barriers to foreign direct investment can contribute to technology transfer and job creation but it can also allow transnational risk commodity corporations (TRCCs) to enter new markets, increasing the diversity and volume of risk commodities available. This generates intensified competition that can drive prices down increasing the affordability and demand for risk commodities,^5^ which together affects individual consumption behaviours and ultimately exposure to NCD risk factors.
- Governance by feedback and self-organization, with behaviours emerging spontaneously from the feedback structures between agents and elements. Neoliberally orientated policy actors have developed institutions like the World Trade Organization, the rules of which can deepen policy actors’ adherence to neoliberal norms. From this emerges persistent patterns of neoliberally-driven policy decisions, for example, prioritization of unobstructed international trade in risk commodities over health objectives.
- Nonlinearity, such that an effect is rarely proportional to its cause and is often due to multiple considerations influencing decision-making over time. For example, increasing pressure from public health advocates may increase policy-makers’ motivation to reduce population exposure to risk commodities up to the point when economic goals are perceived to be compromised. Here the desire to comply with economic norms in policy-making begins to dominate and despite further increase in pressure from health advocates, this no longer motivates policy-makers’ decisions.
- Historical-dependency, where institutions and societal norms take particular trajectories, and many actions cannot be undone or take a very long time to undo. For example, irreversible historical processes may explain why certain TRCCs have exerted significant political influence in many countries but not managed to so do in others.
- Adaptation and evolution of abilities and behaviours over time. For example, as the rules and accepted norms of engagement between tobacco companies and governments have changed and tobacco control legislation has tightened in many countries, tobacco companies have been forced to adopt new strategies to ‘chill’ regulatory development, including the use of international trade and investment treaties.
- Counterintuition. Cause and effect are often very distant in time and space, but people tend to focus on addressing only local causes. Public health practitioners often focus on changing individual behaviour to reduce consumption of risk commodities through knowledge sharing and education programs and are less likely to pursue higher level supply-side regulations for example interventions in agricultural production and food processing to promote healthy diets.

This suggests that addressing NCD policy coherence challenges that have emerged from compliance with neoliberal norms, such as international investment policy promoting TRCC entry into a country, entering into a new trade deal, or the involvement of commercial actors in policy-making may be complex system problems, but how can a systems approach be operationalized? System dynamics and soft system methodologies (SSM) offer two of the most sophisticated perspectives and methodological approaches. In SSM models of key elements (entities, structures and viewpoints) of a complex human activity problem are developed from different worldviews to learn about and guide discussion on how to improve a situation.^6^ System dynamics modelling (SDM) focuses on understanding the relationship between system elements by incorporating feedback and time delays that help capture the ‘dynamic, evolving and interconnected’ nature of complex system problems.^2^ While SSM offers a promising process for collective learning about problems related to the risk commodity environment, we focus here on SDM methods since we believe exploring the dynamics between system elements will also be important to improve understanding of how the risk commodity environment has emerged and to identify effective points of intervention. SDM attempts to define the causal structure of a complex problem by engaging stakeholders across relevant sectors in the co-production of a shared mental model (SMM), visualizing the system elements most relevant to the problem and the nature of their connection in causal loop diagrams. Consequently, using the SDM process for developing a shared understanding of how and why specific, bounded risk commodity-related NCD problems arise from neoliberal policy-making norms could also be useful in itself to reduce the ‘tension’ and ‘distance’ that exists between health, economic and agricultural sectors, as described by Lencucha and Thow.^1^ The SMM can also help identify potential high-leverage interventions within the system and inform discussion about their possible intended and unintended impacts. Where feasible, a SMM can be translated into a simulation model to estimate the potential consequences of different interventions, helping to identify effective and complementary policy levers across sectors which may contribute to the development of a more coherent approach to NCD prevention. For example, a simulation model may be useful to estimate the impacts of different interventions including a sugar sweetened beverage tax or an agricultural subsidy. Methods from the field of system dynamics have already been used to model, for example, tobacco control policies.^7,8^

While SDM methods could be used to better understand how NCDs have emerged from neoliberal policy affecting the risk commodity environment and to begin to identify more coherent policy options, the second half of this commentary focuses on how a systems thinking approach more broadly may be useful in conceptualizing the related but different complex problem of *how* to translate a recommended approach into policy. Meadows outlines a core set of eleven places to intervene in a complex system, each with varying degrees of effectiveness. One of these is ‘system rules’; incentives, punishments and constraints.^9^ Lencucha and Thow described how the Guidelines for the Implementation of Article 5.3 of the Framework Convention on Tobacco Control introduced enforceable rules of engagement between policy-makers and the tobacco industry. Changing system rules is considered to be relatively effective for contributing to system evolution.^9^ Paradigms, however, determine the system’s rules, as well as its goals, parameters and structure.^9^ While very difficult to achieve, intervention at the paradigmatic level of a system is therefore highly effective and may be the only way to force the revolution likely necessary to bring about coherent policy action across sectors for the prevention of NCDs.

As illustrated by Lencucha and Thow, the neoliberal paradigm has infiltrated and dominated public policy-makers’ values, reasoning and judgment almost without contest for over half a century. Neoliberalism has set the system goal as economic growth at almost any cost, determined that risk commodity corporations play a powerful role in shaping public policy and has shaped the rules of our institutions to privilege business interests over public health in public policy decisions. The public health community has clearly described the detrimental impacts on health and social outcomes and equity, but whilst public health and social advocates have managed some tweaks around the edges of risk commodity impact (eg, introducing sugar taxes, advertising bans and health warning labels), establishing effective and sustainable policy change may need paradigmatic transformation. In this, public health advocates have the tall task of needing to define an attractive enough alternative and gaining the requisite political support for its implementation. While we, like other public health academics, are no experts in catalyzing paradigmatic transformation, we suggest that a strategy for change will require a transdisciplinary approach between health, social, environmental and new economics advocates in order to establish both an appealing and inspiring alternative to neoliberalism and to capture the imaginations of economic, agricultural and other sector policy-makers. Raworth’s Donut of social and planetary boundaries framework (developed with the help of systems thinking) provides the foundations of a promising alternative paradigm.^9^ Instead of economic growth as a primary goal, the Donut economic model aims to meet the needs of all within the means of the planet.^10^

Convincing policy-makers to adopt a new economic paradigm better suited to 21st century challenges will likely require public health advocates to move beyond promoting the narrow public health benefits of NCD prevention and prove that a paradigmatic shift would have a multiplicity of sustained benefits across sectors including the economy and environment. The planetary health perspective recognizing the interdependencies of human health and natural systems offers an example of systems-based transdisciplinary research identifying interventions to reduce exploitative use of resources linked to unsustainable consumption and production patterns that reduce and prevent risks to both human health and the environment.^11^ For example, both ‘environmental and health benefits are possible by shifting current Western diets to a variety of more sustainable dietary patterns.’^12^ To translate this kind of research into effecting a paradigmatic shift triggering coherent policy action, the public health research community will need to move beyond ‘policy-maker engagement’ rhetoric and learn how to be effective advocates and even campaigners, for example by harnessing the momentum of relevant current political and social movements. Working with – and learning from – the global climate movement for example, offers such an opportunity that has yet to be fully realized.^13,14^

## Acknowledgements

Penelope Milsom received doctoral funding from the Wellcome Trust [203286/Z/16/Z].

## Ethical issues

Not applicable.

## Competing interests

Authors declare that they have no competing interests.

## Authors’ contributions

PM and HW designed the commentary and PM drafted the manuscript. All authors contributed to critical revisions of the manuscript.

## Authors’ affiliations

^1^Department of Global Health and Development, Faculty of Public Health and Policy, London School of Hygiene and Tropical Medicine, London, UK. ^2^College of Medicine and Health, University of Exeter, Exeter, UK.
